# Bacterial community structure in rotating biological contactor treating coke wastewater in relation to medium composition

**DOI:** 10.1007/s11356-019-05087-0

**Published:** 2019-05-03

**Authors:** Aleksandra Ziembińska-Buczyńska, Sławomir Ciesielski, Sebastian Żabczyński, Grzegorz Cema

**Affiliations:** 1 Environmental Biotechnology Department, Silesian University of Technology, Akademicka 2, 44-100 Gliwice, Poland; 2 Faculty of Environmental Sciences, Department of Environmental Biotechnology, University of Warmia and Mazury in Olsztyn, Słoneczna 45G, 10-719 Olsztyn, Poland

**Keywords:** NGS, Coke wastewater, Biofilm, Bacterial community shift

## Abstract

Biological wastewater treatment using biofilm systems is an effective way to treat difficult wastewater, such as coke wastewater. The information about the structure and the dynamics of this microbial community in biofilm, which are responsible for wastewater treatment, is relevant in the context of treatment efficacy and the biochemical potential to remove various pollutants. However, physico-chemical factors can influence the biofilm community significantly, causing performance disturbances. Therefore, we decided to examine the structure of microbial community in rotating biological contactor (RBC) biofilm during coke wastewater treatment and to investigate the possible shift in the community structure caused by the feeding medium change from synthetic to real coke wastewater. The experiment performed with high-throughput next-generation sequencing (NGS) revealed that bacteria commonly present in wastewater treatment plant (WWTP) systems, responsible for nitrite oxidizing, such as *Nitrospira* or *Nitrobacter*, were absent or below detection threshold, while *Nitrosomonas*, responsible for ammonia oxidizing, was detected in a relatively small number especially after shift to real coke wastewater. This research indicates that medium change could cause the change from autotrophic into heterotrophic nitrification led by *Acinetobacter.* Moreover, biofilm systems can be also a potential source of bacteria possessing high biochemical potential for pollutants removal but less known in WWTP systems, as well as potentially pathogenic microorganisms.

**Electronic supplementary material:**

The online version of this article (10.1007/s11356-019-05087-0) contains supplementary material, which is available to authorized users.

## Introduction

Biological wastewater treatment is a universal and effective method for purification of the majority of liquid wastes. However, some industrial wastewaters, such as coke wastewater, are difficult to be treated with these methods. For coke wastewater, the presence of recalcitrants such as phenols, cyanides, thiocyanides, or heavy metals can interrupt the treatment procedure performed with activated sludge (Pal and Kumar 2014). The microbial cooperation of nitrogen removal bacteria with the other groups of microorganisms present in the wastewater treatment plant (WWTP) community results in efficient wastewater treatment. Such microbial interactions are modeled by physico-chemical parameters influencing the technological systems especially in biological wastewater treatment. The structure and the dynamics of this microbial community can be influenced by external factors such as pH, temperature, substrate, and oxygen concentration (Cydzik-Kwiatkowska and Zielińska 2016). One of the factors influencing bacterial community during wastewater treatment is the wastewater composition (Cydzik- Kwiatkowska et al. 2012; Zhang et al. 2018). The feeding medium change (from municipal to industrial or from synthetic to real wastewater) can cause a drastic shift of the bacterial community structure and it can influence dramatically nitrogen and recalcitrant removal. The feeding medium shift, highly dangerous for activated sludge bacteria, can be led in biofilm-based systems with the relatively lower possibility of bacterial community damage because of the high amount of extrapolimeric substances (EPS) protective against harmful physico-chemical factors influencing the biocoenosis. Moreover, biofilm-based systems are recognized to be an excellent model for ecological relationships research among bacteria in the community due to their direct contact and relatively closer location to each other (Cydzik-Kwiatkowska et al. 2012; Zhang et al. 2018).

Technological systems based on biofilm, to which rotating biological contactors (RBCs) belong, are regarded as a promising technology for treating harmful wastewater. The advantages of such systems are higher biomass concentration, small size, and relatively high efficacy (Cortez et al. 2008; Duque et al. 2014). As such, it provides an excellent opportunity to study numerous bacterial groups present in biofilm communities (Ciesielski et al. 2010). Although there are studies showing the composition of microbial communities in the biofilm of RBC, there is a lack of detailed information about microorganisms responsible for coke wastewater treatment and their community shift while feeding medium change.

Therefore, the main goal of this work was to examine the structure of microbial community in RBC biofilm during coke wastewater treatment and to investigate the possible shift caused by the feeding medium change from synthetic to real coke wastewater. Total bacterial community description was performed using high-throughput next-generation sequencing (NGS) method in order to present a total picture of the community shift.

## Materials and methods

### Experiment settings and biofilm sampling

**Fig. 1:**
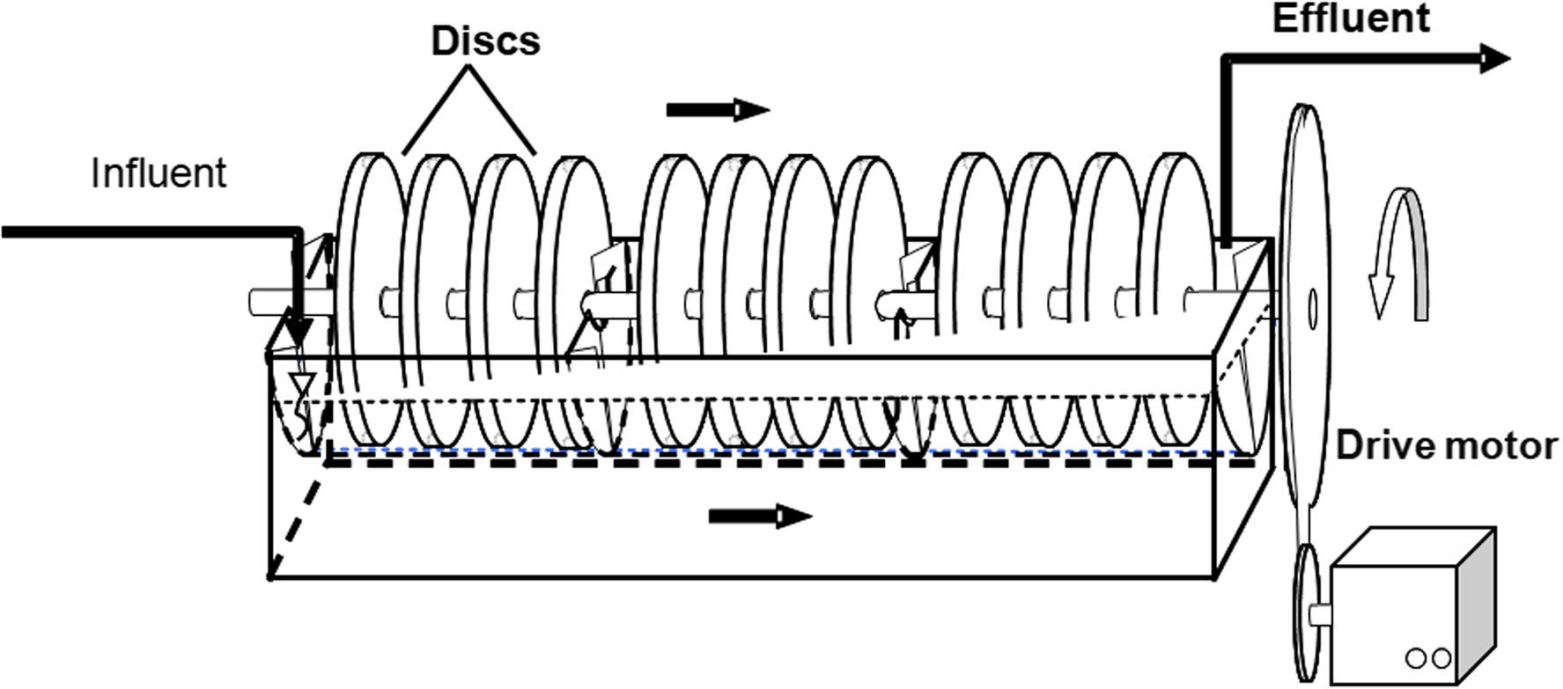
The scheme of rotating biological contactor used in the study (Cema et al. 2016)

The experiment was performed in the laboratory scale rotating biological contactor (Fig. 1). The RBC consisted of three chambers with 12 disks (a group of four per chamber). The disk diameter and total disk surface were equal to 0.225 m and 2.61 m^2^, respectively. The immersion of disks was around 41% and the working volume of RBC was 0.014 m^3^. To prevent algal growth and maintaining a stable temperature, the RBC unit was covered by polystyrene foam. The temperature during the experiment was kept at an average level of 20.2 ± 2.2 °C. The pH in the inflow did not exceed 8.3 throughout the course of the experiment.

The experiment was operated for 823 days and divided into two periods. In the first experimental period, the RBC was fed with artificial coke wastewater for 719 days (days 0–719). The second experimental period involved treating the RBC with real coke wastewater from “Jadwiga” coke plant in Zabrze (Southern, Poland) for 104 days (days 719–823). A more detailed description of the experimental system, together with the composition of synthetic and real coke wastewaters used for the experiments, is described elsewhere (Cema et al. 2016).

Biofilm samples (volume of 20 mL) were collected from all RBC disks, mixed together to create a composite sample, and frozen at − 45 °C until further processing.

### DNA extraction

Genomic DNA was extracted from 0.2 g of semi-dry biofilm samples collected at days 632 and 823 of the process. DNA was isolated using the FastDNA Spin Kit for soil (MP Biomedicals, USA) as per the instructions of the manufacturer. Qubit 2.0 Fluorometer (Invitrogen, USA) was used to obtain accurate DNA quantification. The purified DNA was suspended in 100 μL of deionized, DNAase free water and stored at − 20 °C.

### Library preparation and Illumina sequencing

The microbial community was analyzed by the amplification of the V3–V4 regions of the 16S rRNA gene, performed with S-D-Bact-0341-b-S-17 (5′ TCGTCGGCAGCGTCAGATGTGTATAAGAGACAGCCTACGGGNGGCWGCAG 3′) and S-D-Bact-0785-a-A-21 (5′ GTCTCGTGGGCTCGGAGATGTGTATAAGAGACAGGACTACHVGGGTATCTAATC 3′) Illumina-recommended primers (Klindworth et al. 2013). Amplicons were indexed using Nextera® XT Index Kit according to the manufacturer’s instructions. DNA was sequenced on an Illumina MiSeq instrument using a 2 × 250 paired-end mode.

### Bioinformatics analyses

The sequencing results were recorded as FASTQ files and uploaded to the MetaGenome Rapid Annotation Subsystems Technology (MG-RAST) server where normalization, transformation, and alpha diversity measurement were conducted (Meyer et al. 2008). Each file underwent quality control (QC), which included quality filtering (removing sequences with ≥ 5 ambiguous base pairs) and length filtering (removing sequences with a length ≥ 2 standard deviations from the mean). The 16S rRNA datasets are available at MG-RAST under accession numbers 4,629,653.3 (632) and 4,629,653.3 (823). Taxonomic differences were analyzed using the Statistical Analysis of Metagenomic Profiles (STAMP v. 2.1.3) (Parks and Beiko 2010). Statistically significant differences between samples were identified by Fisher’s exact test combined with the Newcombe-Wilson method for calculating confidence. A column chart comparing the relative abundances of each class was generated using Microsoft Excel.

## Results and discussion

### Physico-chemical changes after real wastewater introduction

From the point of wastewater treatment efficacy, nitrogen removal is a crucial process and the researches on the nitrogen removal communities treating various sewage types are commonly performed (Kowalchuk et al. 1997; Rotthauwe et al. 1997; Nicolaisen and Ramsing 2002; Wertz et al. 2008; Vanparys et al. 2007; Attard et al. 2010).

**Fig. 2:**
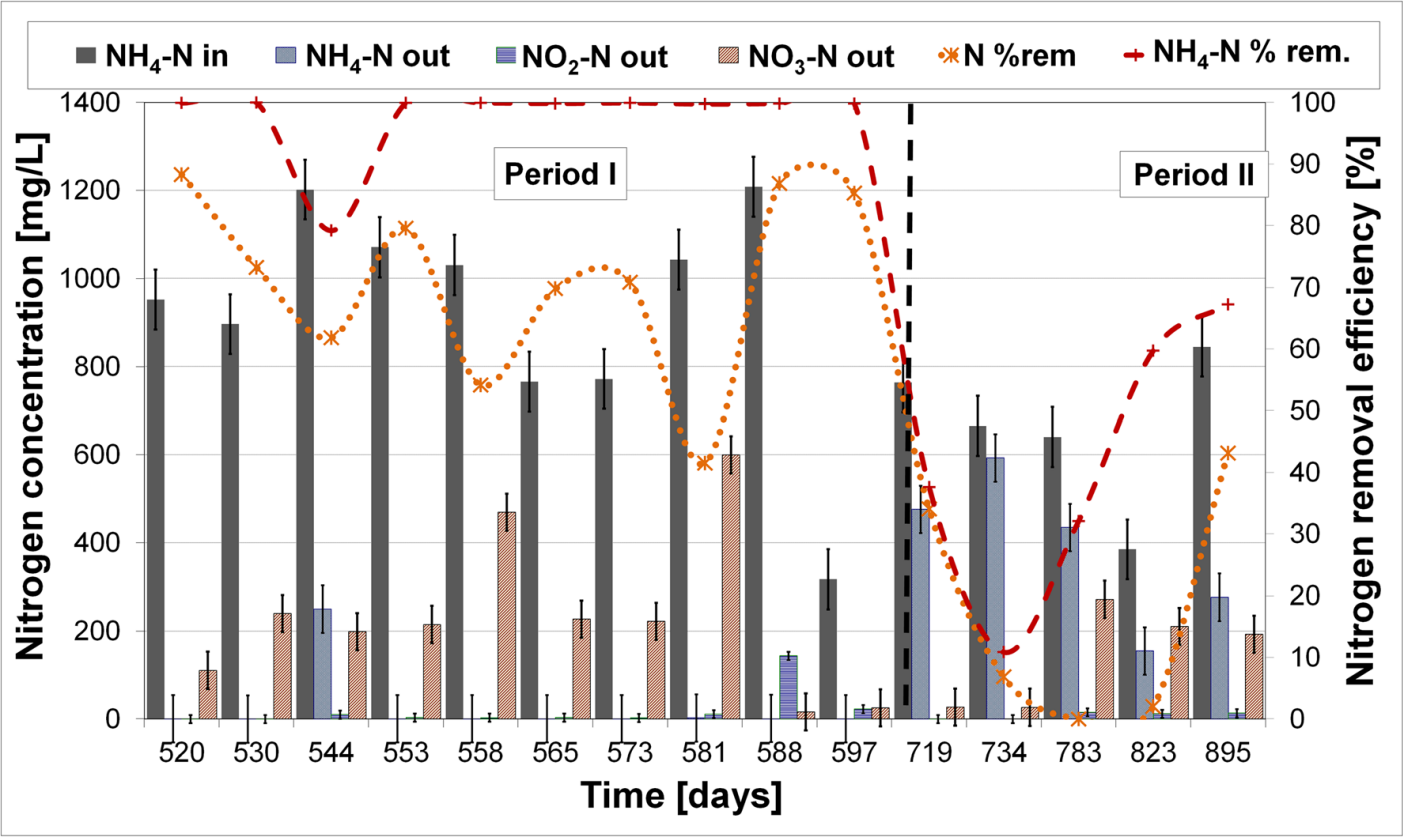
Ammonia nitrogen removal performance in RBC during the total length of the experiment; period I—synthetic coke wastewater treatment; period II—real coke wastewater treatment (in - inflow, out - outflow, rem. - removal)

In the case of coke wastewater, the situation is more complex. This sewage is regarded as difficult for biological treatment; thus, the bacterial community able to treat this wastewater needs to be specialized, easily adapting and relatively diverse in the case of the necessity of recalcitrant removal. The RBC biofilm bacterial community in this experiment was performing stable nitrogen removal from the beginning of the experiment on synthetic coke wastewater for 719 days. After this period, the real coke wastewater was introduced to the system and the experiment was performed further as it has been described in detail by Cema et al. (2016). Figure 2 presents the nitrogen removal in RBC system during the shift of the feeding medium change.

From 520th day of research and during period I of research, the average nitrogen concentration in the influent was equal to 930.5 ± 263.0 mg/L. The removal efficiency in RBC was 71.1 ± 20.3% with very efficient and stable ammonium nitrogen removal equal to 97.8 ± 6.5% (Table 1). Also, the phenol removal was very efficient at 99.7 ± 0.2% with an influent concentration equal from 175.6 to 32.3 mg/L. After the shift to the real coke wastewater, the sudden process breakdown was observed (Fig. 2). The nitrogen removal drops from over 80% at the end of the period I to almost 0 at the beginning of period II. Also, phenol concentration was fluctuating. The rebuild of the nitrogen removal took over 150 days (the results of nitrogen fluctuation in the reactor were described in detail in Cema et al. 2016).

**Table 1:** Average values of nitrogen concentration, removal efficiency, and nitrogen load and removal rate during the shift of the feeding medium change (data presented with standard deviation)

	N in (mg/L)	N out (mg/L)	N removal efficacy (%)	NH_4_-N removal efficacy (%)	N load (g/m^2^/day)	N removal rate (g/m^2^/day)
Synthetic wastewater	930.5 ± 263.0	278.3 ± 178.5	71.1 ± 20.3	97.8 ± 6.5	1.4 ± 0.5	1.0 ± 0.4
Real wastewater	660.4 ± 174.7	540.7 ± 132.9	17.2 ± 19.9	41.5 ± 22.6	0.7 ± 0.4	0.1 ± 0.2

### The microbial community shift after real wastewater introduction

The aim of this study was to present the shift of the stable performing bacterial community after the medium change from synthetic to real coke wastewater. In order to analyze the qualitative changes of the microbial community structure, we compared the 16S rRNA gene amplicons data derived from two samples taken during process performance strictly before (632 days) and after the medium change on day 823 (the last sample collected in the system fed with real wastewater). Alpha diversity values for samples taken at days 632 and 823 were 29.48 and 21.23, respectively. Rarefraction curves for each of the samples were almost asymptotic suggesting that the majority of taxonomic diversity was uncovered. Both alpha diversity and rarefraction curves were examined using MG-RAST (Supp. 1, Fig. 1). Bacteria constitute 98.6% of the total community in sample 632 and 98.8% in sample 823. Such a result is obtained in most research performed on the bacterial communities in wastewater systems (Ma et al. 2015).

**Fig. 3:**
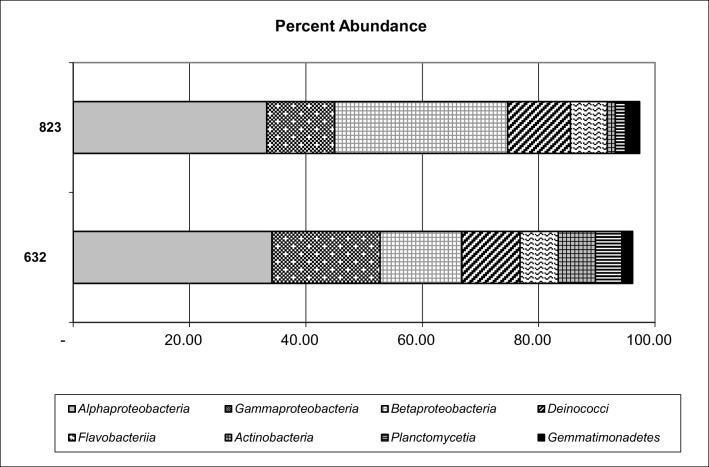
Class level affiliations assigned to reads with 16S rRNA genes in analyzed samples. Only classes with the relative abundance higher than 1.0 % are given

**Fig. 4:**
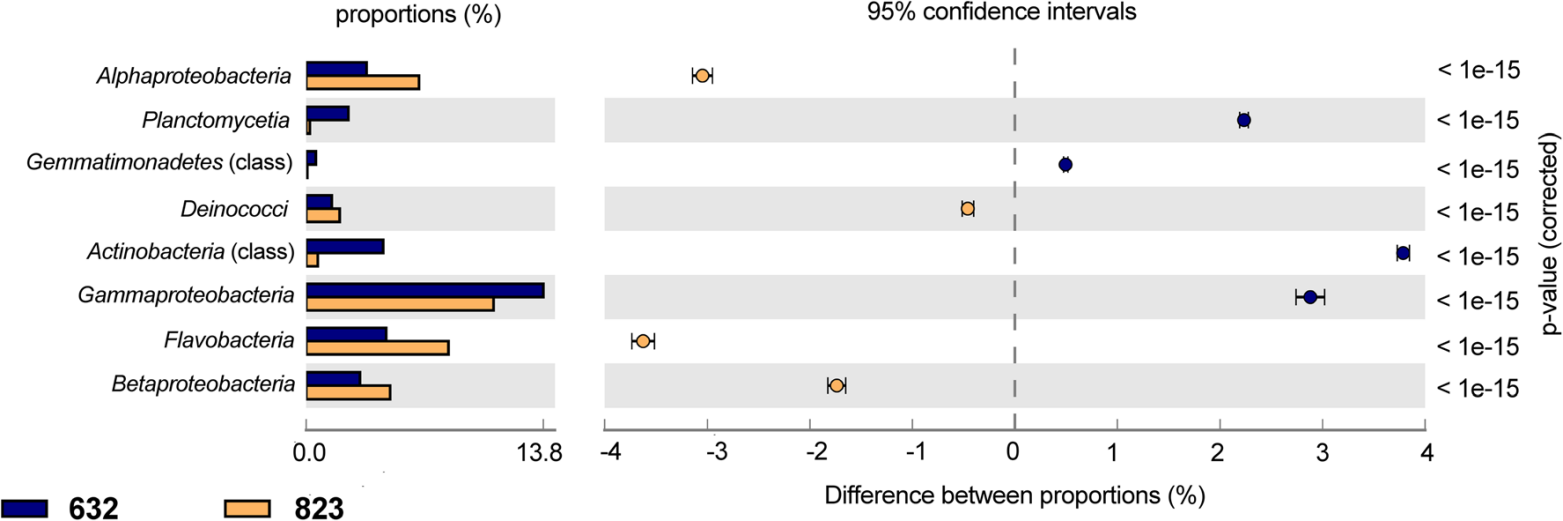
Statistical analyses of taxonomic profiles for the 632 and 823 samples. Classes overrepresented in the 823 sample have a negative difference between proportions (orange dots); those overrepresented in the 632 sample have a positive value difference between proportions (blue dots). Features with a *q* value of 0.05 were considered significant

At class level, 36 bacterial classes were found in 632 sample, and 33 bacterial classes in sample 823. The proportions of the 8 major classes are presented in Fig. 3. Reads affiliated to *Alphaproteobacteria*, *Betaproteobacteria*, and *Gammaproteobacteria* constituted the majority of the communities. STAMP comparison at the class level shows significant differences between analyzed samples (*P* = 95%, Fig. 4). *Gammaproteobacteria*, *Actinobacteria*, *Planctomycetia*, and *Gemmatimonadetes* were overrepresented significantly in sample 632, whereas *Alphaproteobacteria*, *Betaproteobacteria*, *Flavobacteria*, and *Deinococci* were overrepresented in sample 823 (Fig. 4). The predominance of *Betaproteobacteria* in sample 823, 29.74 % vs. 14.08 % in sample 632, may be explained with the removal of betaproteobacterial representatives from the system, due to a change in feeding medium, which in turn caused a decrease in nitrogen removal efficacy. In most cases, *Betaproteobacteria* are found to be predominant (Ma et al. 2015; Felföldi et al. 2010), but in WWTPs dealing with phenol-rich wastewater (such as coke wastewater), *Alphaproteobacteria* and *Gammaproteobacteria* were predominant (Figuerola and Erijman 2007; Wang et al. 2012) and *Alphaproteobacteria* can exceed *Gammaproteobacteria* (Xia et al. 2010). These results may also be attributed to the treatment method used. As it has been stated (Felföldi et al. 2010), *Proteobacteria* are predominant in most cases in WWTP systems, both in biofilm and activated sludge, but it is possible that the ratio of the particular *Proteobacteria* classes differs significantly when the analysis is performed with DNA sequencing or the other method (such as T-RFLP).

Research revealed the decrease of the *Actinobacteria* and *Planctomycetia* members during the process. Their abundance was reduced from 6.36 to 1.40% in the case of *Actinobacteria* and from 4.54 to 1.74% in the case of *Planctomycetia.* These results also underline the difference between wastewater systems based on activated sludge and biofilm. Moreover, these results could be linked with the decrease of nitrogen removal efficacy which in biofilm systems can be performed at large scale by Anammox process performed by *Planctomycetia*. In case the of activated sludge classes, *Firmicutes*, *Chlorobi*, and *Chloroflexi* are often dominant in the system (Felföldi et al. 2010; Ma et al. 2015; Wang et al. 2015), while for biofilm, these classes are not recognized as the main groups.

As it was previously stated by Cydzik-Kwiatkowska and Zielińska (2016) and Nascimento et al. (2018) also in the case of this research, the change of feeding wastewater can be the reason of the class proportion shift. It could be caused by the concentration changes in organic matter or recalcitrant substances.

Bacterial diversity and abundance were analyzed more specifically at the genus level (Tables 2 and 3). Table 2 showed genera which number increased during the process of coke wastewater treatment. The most significant change can be observed for *Acinetobacter*, *Parabacteroides*, *Riemerella*, and *Pseudomonas*. The number of these genera increased respectively: 567, 77, 36.17, and 18.17 times, during the experiment time when the medium was changed from synthetic to real coke wastewater. These results support the previous research of Liu et al. (2015). They have isolated *Acinetobacter* from activated sludge treating coke wastewater which was able to remove ammonium, nitrite, and nitrate with a very high efficacy*.* Our results can support the thesis that real medium causes bacterial community change, seen especially in the group of nitrifiers, to prevent nitrogen removal breakdown. The activity of autotrophic nitrogen-removing bacteria in RBC (such as *Nitrosomonas*, Table 3) was suppressed by the harmful real coke wastewater directed to the system and such change created environment suitable for heterotrophic nitrogen-removing bacteria, such as *Acinetobacter.* Also, the increase of *Pseudomonas* bacteria number supports this thesis due to the fact that both *Pseudomonas* and *Acinetobacter* are common coke wastewater treatment plant inhabitants and they are also able to remove thiocyanates as a sole carbon source (Boucabeille et al. 1994; Huang et al. 2013). It is worth mentioning that heterotrophic nitrifiers have attracted increasing attention as this group of bacteria grow faster and is less sensitive towards high loads of ammonium and organic matter than autotrophic nitrifiers (Liu et al. 2015). It is known that heterotrophic nitrification could be performed under a wide range of C/N ratio (Yang et al. 2017), but the research in this field are still scarce; thus, it would be reasonable to undertake such study especially in the field of coke wastewater treatment, where this process seems to be common (Liu et al. 2015; Lü et al. 2012; Yang et al. 2017).

**Table 2:** The list of bacterial genera that increased during process performance (only genera with the relative abundance higher than 0.1% are shown)

Genus	632 sample	823 sample	Change (fold)
*Acinetobacter*	0.01	5.67	567.00
*Parabacteroides*	0.02	1.54	77.00
*Riemerella*	0.06	2.17	36.17
*Pseudomonas*	0.06	1.09	18.17
*Bacteroides*	0.27	0.94	3.48
*Candidatus Solibacter*	0.23	0.57	2.48
*Bradyrhizobium*	0.56	1.32	2.36
*Alcaligenes*	0.37	0.82	2.22
*Myroides*	0.92	1.58	1.72
*Elizabethkingia*	0.37	0.51	1.38
*Prolixibacter*	0.49	0.66	1.35
*Deinococcus*	2.10	2.64	1.26

**Table 3:** The list of bacterial genera that decreased during process performance (only genera with the relative abundance higher than 0.1% are shown)

Genus	632 sample	823 sample	Change (fold)
*Microbacterium*	1.23	0.01	123
*Planctomyces*	2.82	0.04	70.5
*Gramella*	0.56	0.01	56
*Prosthecobacter*	1.35	0.03	45
*Subtercola*	0.58	0.02	29
*Sphingobacterium*	0.74	0.03	24.66
*Pirellula*	0.53	0.023	23.04
*Leifsonia*	0.8	0.05	16
*Glaciibacter*	1.02	0.14	7.28
*Flavobacterium*	1.09	0.18	6.05
*Isosphaera*	0.7	0.12	5.83
*Hyphomicrobium*	0.54	0.1	5.4
*Salinibacterium*	0.62	0.15	4.13
*Hymenobacter*	1.74	0.62	2.8
*Chthoniobacter*	1.09	0.53	2.05
*Cytophaga*	2.48	1.3	1.9
*Nitrosomonas*	1.34	0.93	1.44

The increase of *Parabacteroides* seems to be a natural process, because this anaerobic bacterium and its close relatives have been already identified in the biofilm system performing nitrogen and phosphorus removal (Feng et al. 2013); thus, it could be suspected that they are the natural biofilm inhabitant and the conditions in the system caused by the medium change was suitable for their multiplication. Their increase in the biofilm could be explained with their anaerobic metabolism which locates these bacteria in the bottom of the biofilm. *Parabacteroides* was protected against the harmful real coke wastewater influence by the upper layers of the biofilm in which aerobic nitrifiers were located.

Wastewater treatment plants are a potential source of pathogenic bacteria that can be spread not only via water and soil but they also can be aerosolized, becoming a source of airborne infections (Yang et al. 2019). NGS-based studies of pathogens present in WWTP communities as well as their presence and quantification in the effluent have already been recognized as a good solution for bacterial diseases prevention method (Lu et al. 2015). As WWTP bacterial communities are highly diverse, they can be a source not only of the common pathogens such as *Bacillus* (*B. cereus*) or *Escherichia coli* (Uhrbrand et al. 2017) but they can function as a reservoir of less known but comparably dangerous microorganisms. This study revealed the presence of several bacteria recognized as pathogenic but relatively less known and common.

The genus *Riemerella* is mainly known as a bird disease agent (mainly pigeons and ducks) (Segers et al. 1993; Rubbenstroth et al. 2013). But it was also found in wastewater treatment systems with activated sludge (Meli et al. 2016). This result supports the statement that all types of WWTPs could be the reservoirs of pathogenic bacteria.

The number of bacterial genera, *Microbacterium*, *Planctomycetes*, *Gramella*, *Prosthecobacter*, *Subtercola*, *Sphingobacterium*, and *Pirellula* and *Leifsonia*, decreased in sample 823 more than tenfold in comparison with sample 632 (Table 3).

Although *Microbacterium* is known to be a pathogenic bacteria, according to Chung et al. (2016), *Microbacterium* was isolated from the textile wastewater and able to degrade polyvinyl alcohol together with the other isolated strain of *Paenibacillus.* Moreover, it is also described as sulfamethoxazole degrader (Bouju et al. 2012). Thus, it could be suspected that real coke wastewater caused the decrease of these bacteria due to their higher sensitivity (as potentially pathogenic bacteria), but in the first part of the experiment on the synthetic wastewater, this bacterium could be responsible for recalcitrant and other wastewater compounds removal.

The decrease of *Planctomycetes* as the representatives of ammonium removal bacteria seems to be justified also at this taxonomical level. High toxic load in the feeding medium was the probable reason why these bacteria number was reduced. This change probably caused the change of autotrophic nitrogen removal into heterotrophic. It is worth mentioning that *Pirellula*, in which the number also decrease during the experiment, belongs to marine *Planctomycetes* (Glöckner et al. 2003) but probably is not linked with anaerobic ammonia oxidation; however, it could be suspected that the mechanism of harmful influence of real coke wastewater in both microorganisms is similar.

*Gramella*, a representative of *Bacteroidetes*, possesses *NosZ* genes linked with denitrification (Jung et al. 2013), so it could be suspected that in this research *Gramella* was responsible for denitrification but its number decreased due to the harmful real coke wastewater influence on the nitrogen removal bacteria. The same factor caused the decrease of *Subtercola* which according to the patent of Vanotti et al. (2009) is one of 16 critical genera in the activated sludge performing nitrification of high ammonium concentration at low temperature; thus, it is possible that the fluctuations of ammonia nitrogen concentration and that higher than in the first part of the experiment temperature of the real wastewater were the factors causing the decrease of these bacteria number.

According to the previous research, there are some representatives of *Prosthecobacter (P. algae* sp. nov.) isolated from activated sludge able to nitrate reduction to nitrite (Lee et al. 2014); thus, it could be suspected that bacteria belonging to this genus could be responsible for nitrogen removal processes in wastewater treatment and real coke wastewater was also the factor causing their number decrease.

According to previous statements, *Sphingobacterium* is relatively ubiquitous. These bacteria have been isolated from Antarctic soil, compost, and clinical samples also with opportunistic infections (Holmes et al. 1982; Yabuuchi et al. 1983; Shivaji et al. 1992; Kim et al. 2006). Also, *Leifsonia* and its close relatives were isolated from the environmental samples such as sewage sludge compost (Vaz-Moreira et al. 2008), soil (Madhaiyan et al. 2010), and water (Han et al. 2013). In the case of Han et al.’s (2013) research, these bacteria caused septicemia. Decrease of these genera number could be caused by high toxic load in real coke wastewater, but still, its presence in the wastewater treatment system underlines the fact that wastewater and systems for its treatment can be a pathogen reservoir.

## Conclusions

Molecular approach enables to show the change in bacterial community structure driven by many environmental factors. The feeding medium change influences the wastewater treatment efficacy; thus, it could be assumed that it also influences the bacterial community composition. This analysis revealed that bacteria usually recognized as commonly present in WWTP systems such as *Nitrospira* or *Nitrobacter* in this case were absent or below detection threshold. From the group of bacteria typically recognized as nitrogen-removing, only *Nitrosomonas* was above the detection threshold and its relative abundance decreased during the experiment. Instead, there is a large number of bacteria, less known in the field of nitrogen removal in wastewater treatment, which probably can lead several processes belonging to nitrogen cycle in the WWTP systems, such as the heterotrophic nitrification (*Acinetobacter* sp.) or denitrification (*Gramella* sp.). Particularly, heterotrophic nitrification and bacteria performing this process seem to be interesting topic as such microorganisms have been already found and described in coke wastewater plants as important players in nitrogen cycle. Also, the other genera such as *Pseudomonas* sp. or *Microbacterium* sp., in which its presence in WWTP systems can be surprising, may deal with a myriad of recalcitrant effectively.

This research revealed a high number of pathogenic bacteria and the other microorganisms regarded as potentially pathogenic (such as *Leifsonia* or *Sphingobacterium*). These results indicated the necessity to monitor not only the WWTP systems but also wastewater effluent for the possibility of potentially pathogenic bacteria presence for the safety reasons.

These results supports the thesis that in order to describe and understand function and changeability of complex bacterial communities (not only in biofilm) dealing with difficult types of wastewater, polyphasic approach with a wide range of molecular biology tools is needed. Paying attention to bacteria able to degrade toxic substances which can also be able to remove other compounds (such as nitrogen or phosphorus) is also relevant.

## Electronic supplementary material

ESM 1 (DOCX 187 kb)
