# Supplementary figures and images for: Bacterial community structure in rotating biological contactor treating coke wastewater in relation to medium composition
Source: Environ Sci Pollut Res Int. 2019 May 3;26(19):19171–9. doi: 10.1007/s11356-019-05087-0 (PMC6594990; doi:10.1007/s11356-019-05087-0)

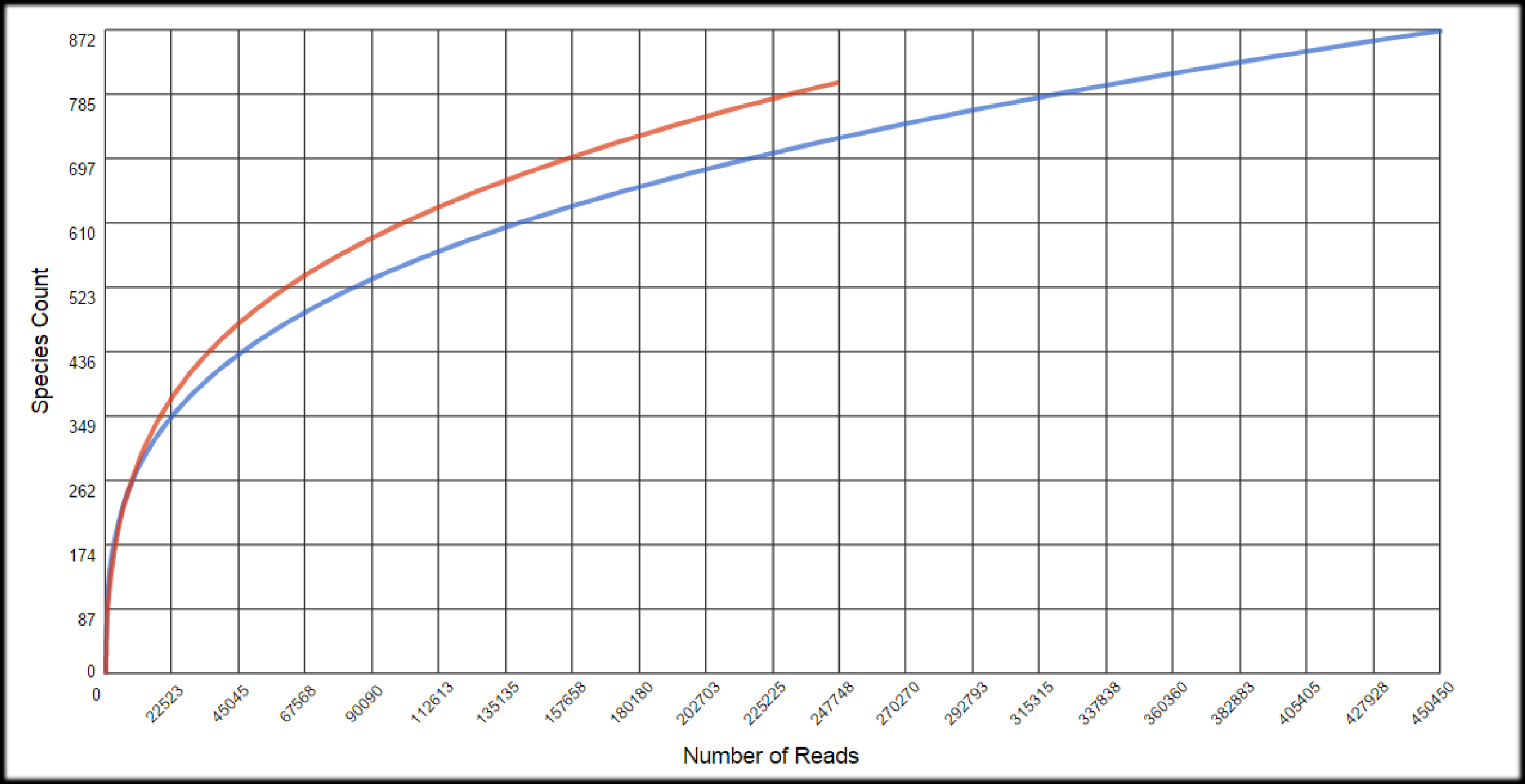


Supp. 1 Fig. 1. Rarefraction curves for the analyzed samples.

Supplement: Supplementary file 1 — (DOCX 187 kb) [file 11356_2019_5087_MOESM1_ESM.docx]
